# Free Energy and Virtual Reality in Neuroscience and Psychoanalysis: A Complexity Theory of Dreaming and Mental Disorder

**DOI:** 10.3389/fpsyg.2016.00922

**Published:** 2016-07-15

**Authors:** Jim Hopkins

**Affiliations:** Research Department of Clinical Educational and Health Psychology, University College LondonLondon, UK

**Keywords:** free energy, complexity, emotional conflict, memory consolidation, synaptic pruning, dreaming, mental disorder

## Abstract

The main concepts of the free energy (FE) neuroscience developed by Karl Friston and colleagues parallel those of Freud's Project for a Scientific Psychology. In Hobson et al. (2014) these include an innate virtual reality generator that produces the fictive prior beliefs that Freud described as the primary process. This enables Friston's account to encompass a unified treatment—a complexity theory—of the role of virtual reality in both dreaming and mental disorder. In both accounts the brain operates to minimize FE aroused by sensory impingements—including interoceptive impingements that report compliance with biological imperatives—and constructs a representation/model of the causes of impingement that enables this minimization. In Friston's account (variational) FE equals complexity minus accuracy, and is minimized by increasing accuracy and decreasing complexity. Roughly the brain (or model) increases accuracy together with complexity in waking. This is mediated by consciousness-creating active inference—by which it explains sensory impingements in terms of perceptual experiences of their causes. In sleep it reduces complexity by processes that include both synaptic pruning and consciousness/virtual reality/dreaming in REM. The consciousness-creating active inference that effects complexity-reduction in REM dreaming must operate on FE-arousing data distinct from sensory impingement. The most relevant source is remembered arousals of emotion, both recent and remote, as processed in SWS and REM on “active systems” accounts of memory consolidation/reconsolidation. Freud describes these remembered arousals as condensed in the dreamwork for use in the conscious contents of dreams, and similar condensation can be seen in symptoms. Complexity partly reflects emotional conflict and trauma. This indicates that dreams and symptoms are both produced to reduce complexity in the form of potentially adverse (traumatic or conflicting) arousals of amygdala-related emotions. Mental disorder is thus caused by computational complexity together with mechanisms like synaptic pruning that have evolved for complexity-reduction; and important features of disorder can be understood in these terms. Details of the consilience among Freudian, systems consolidation, and complexity-reduction accounts appear clearly in the analysis of a single fragment of a dream, indicating also how complexity reduction proceeds by a process resembling Bayesian model selection.

Shortly after starting to analyse his own and his patients' dreams Freud concluded that dreams were produced by the same neurocognitive mechanisms as the neurotic symptoms on which he had previously concentrated. He initially framed this unifying discovery in a “psychology for neurologists”—posthumously published as his *Project for a Scientific Psychology* (Freud, 1974a)—in which he envisaged the brain as operating to minimize free energy (FE). There he noted that “the pathological mechanisms which are revealed in the most careful analysis in the psychoneuroses bear the greatest similarity to dream-processes” (p. 336).

Both dreams and symptoms, Freud now hypothesized, served to defend the conscious self (or ego) by reducing FE from otherwise distressing and functionally disruptive arousals of emotion and conflict. Both did so via the creation of *fictive* experiences and beliefs, forms of virtual reality or phantasy creating an “alienation from reality” that *masked* and *pacified* (or in neuroscientific terms *inhibited*) the arousals. In this he recast a longstanding tradition—exemplified by Kant's (1764/2007) (1764/2007, p. 71) claim that “the deranged person” was “a dreamer in waking”—that linked dreaming with mental disorder.

Freud sought to integrate this tradition with depth psychology, psychiatry, and neuroscience, and it has had many other distinguished advocates in the sciences of the mind since he wrote. The publication of Hobson et al. (2014)—hereafter abbreviated as HHF—seems an important step toward fuller integration. The authors bring the Bayesian neuroscience recently advanced by Karl Friston and his colleagues under the *free energy principle* (Friston, 2010) into a full functional isomorphism with Freud's early formulations. This means that their hypotheses linking dreaming and *complexity* (also described as Bayesian surprise) can naturally be extended to encompass a *complexity theory of mental disorder*^1^.

As advanced here this is the theory that the conflicts and traumas that Freud thought responsible for recourse to phantasy/virtual reality in mental disorder should be seen as forms of neurocomputational complexity, and that mental disorder is the product of such complexity together with the mechanisms that have evolved to reduce it. Although, I emphasize Freud in stating this theory it has consequences for mental disorder that go beyond psychoanalysis, and that are also relevant to psychiatry. In what follows I will try to show that this approach flows directly from the account of complexity that informs HHF, and also to operationalize it with examples from dreaming, attachment, and disorder.

Also it is worth noting at the outset that this theory is *partly* supported by the recent work of Alan Hobson, who followed HHF with the publication of two books (Hobson, 2014, 2015) in which he applies the virtual reality/generative model conception to link dreaming and disorder, urging, for example, that the aminergic-cholinergic balance pivotal for dreaming also unites “a wide range of psychopathological conditions” which “have in common the weakness of the aminergic system.” (Hobson, 2014, p. 51) Indeed, despite his unrelenting criticism of Freud, Hobson now stresses that his current work “takes up the *Project for a Scientific Psychology* exactly where Freud left it in 1895.” (Hobson, 2015, p. 5)

Friston's variational conception of FE derives from the computational work of Geoffrey Hinton and his colleagues (see Dayan et al., 1995), and differs radically from anything Freud could have had in mind. Nonetheless Friston assigns FE the same overall functional role as appears in Freud. In both accounts the brain operates to minimize FE aroused by sensory impingement, with “endogenous [interoceptive] stimuli” a particularly important source. In Freud such impingements reflect “the major needs” or biological imperatives; in Friston they likewise reflect compliance with biological imperatives, predicting departures from homeostasis in a continuously recomputed overall FE-minimizing equilibrium (Pelluzio et al., 2015).

As Freud speaks of these imperatives as creating a “demand for work” to produce “specific actions,” so Friston (2012) speaks of “an imperative to minimize prediction error…through action,” using “[kinematic and proprioceptive] predictions” to produce the “kinematic trajectories” of bodily movement. For both minimization requires the brain to embody a representation or model of the world, including the agent's body (in Freud the “bodily ego”); and for both this requirement is initially met by the innate generation of a *prior* virtual (or phantasy) version of reality, which will subsequently be modified by experience.

Citing evidence that during the final trimester of pregnancy infants spend most of their time in REM, HHF hypothesize that the brain is “genetically endowed with an innate virtual reality generator” whose working “is most clearly revealed in [REM] dreaming.” They thus hold that we are “born with a virtual reality model” of what we will subsequently discover to be the causes of sensory impingement. As well as innate this model is intrinsically predictive, and so is “entrained by sensory prediction errors,” particularly during the sensory initialization attending birth, to become “a generative or predictive model of the world,” operating to minimize the free energy generated by the precision-weighted prediction error (PE) that constitutes its sensory input.

This accords with other observations, such as that during the same prenatal period infants' facial expressions indicate both positive (laughter-related) and negative (cry-related) emotions (Reissland et al., 2011, 2013); and again with the apparently essential role of REM sleep in early learning (Dumoulin Bridi et al., 2015; Boyce et al., 2016). So like Freud's *primary process* the HHF *virtual reality generator* is an innate producer of fictive (imaginary) prior belief and/or experience, the process that, as Freud says is “in the apparatus first.” Thus, Freud describes perception, learning, and action as *secondary processes* prepared for by the innate *primary process*, and set in train by the sensory impact of birth. Comparably, HHF describe these as aspects of *active inference*, the process that implement the transformation from virtual reality to generative model.

Hence also in both Freud and HHF, perceptually informed belief and action replace virtual reality in waking life as memory and the wake-sleep cycle become established, thus relegating the unconstrained production of phantasy/virtual reality to dreaming—where, however, it continues to play a role in minimizing FE. In both accounts, therefore, the REM phase of sleep—which although distinct (doubly dissociable) from dreaming nonetheless yields the most frequent dream reports, and those that are “long, vivid, hallucinatory, and bizarre” (Hobson and Friston, 2012, Figure 1)—can be part of the lifelong genetic regulation of neurological functioning, as envisaged in Jouvet (1999).

## Complexity and accuracy

As HHF emphasize, the FE minimized by the brain can be equated with *complexity minus accuracy*. Accuracy is a measure of the success of a model in predicting the sensory impingements that have accumulated over a given period of data collection, and complexity reflects the number of parameters/hypotheses employed in accomplishing prediction and the extent to which they are altered in the course of doing so. From this it follows that FE can be minimized by *increasing* accuracy and/or *reducing* complexity. There is, however, an intrinsic tension in this task.

The data relevant to the minimization of FE encompass those of sensory impingement. As well as the interoceptive impingements mentioned above, these include exteroceptive impingements on sensory surfaces such as the retina and skin, and proprioceptive impingements reflecting muscular and skeletal kinematics and conditions. If we think of these data as points on a graph, and the hypotheses by which the brain's model seeks to predict them as curves drawn to fit the points, then accuracy would be maximized by a curve, however complex, that passed through every data point.

To increase accuracy over a given sample is to approach this goal for the data of that sample; so increasing accuracy characteristically increases complexity as well. To reduce complexity, by contrast, is to seek the *simplest* curve (the simplest or most economic model) for predicting the data overall. Given that any sample is likely to include many data diverging from the overall trend, this must be a simpler curve than maximizing accuracy would determine. The tension between increasing accuracy and reducing complexity thus shows in the way learning that increases accuracy also tends to *overfit* current data, thereby producing excess complexity as well.

## Development, emotion, and conflict

Basic expectations connected with biological imperatives like homeostasis are woven into the functioning of the brainstem. Comprehensive motor responses to the FE produced by these are required for all aspects of thriving and development. From birth such responses are generated by motoric “prototype emotions systems” delineated in Panksepp (1998), which also perform the motivational work that Freud assigned to the drives (see Damasio and Carvalho, 2013). Watt and Panksepp (2009, p. 98) describe these as “sitting over homeostasis proper (hunger, thirst, temperature regulation, pain, etc.)” and “giv[ing] rise to attachment,” which in turn serves as “the massive regulatory-lynchpin system of the human brain” exercising “primary influence over the prototype systems below.”

The prototype systems are organized around the dopaminergic SEEKING system that apparently energizes purposive activity in waking as well as fictive activity in dreaming. They include “positive” (and rewarding) prototype emotions, such as LUST, PLAY, and CARE, and “negative” (and aversive) prototypes, including the proximity- and attachment-regulating system that Panksepp describes in terms of PANIC, SEPARATION DISTRESS, and GRIEF (hereafter PSG), and those for RAGE and FEAR, which PSG in human infants seems to activate.

The link between homeostasis-maintaining response to interoceptive input by these systems and attachment to caregivers is intrinsic, since human infants cannot maintain life without continual care. As discussed in Hopkins (2015), the vigorous operation of the “negative” systems is essential for the honest signaling of urgent need, so in early infancy both “positive” and “negative” systems are directed at the mother. Infants' cries of distress apparently originate in the (RAGE- and FEAR-generating) hypothalamus and PAG, and they in turn activate the maternal PAG in ensuring rapid response (Parsons et al., 2014).

These systems should accordingly play a major role in the overall integration of behavioral responses to PE—including autonomic and other reflexes, Pavlovian and operant conditioning, and the working of emotion and desire—that Pelluzio et al. (2015) describe the generative model as coordinating over early development. And since the “positive” and “negative” systems tend to generate inconsistent kinematic trajectories toward the same original object of attachment; and since they are parts of an overall structure of emotional opponent processing (Craig, 2015, Ch 8); we should expect a significant part of this postnatal co-ordination to involve the regulation of emotional conflict for the ultimate resolution of interoceptive PE.

## Prediction, perception, and waking consciousness

The most salient hypotheses by which the brain/model works to minimize PE are those that *constitute* the conscious perceptual (or perception-like) experiences of both waking and dreaming consciousness. A basic claim of FE neuroscience, taken from Helmholz (1874/1971) is that the brain both actively collects, and also “synthesizes,” the fragmentary and fleeting manifold of impingements that generate FE in waking, so as to re-represent them as *the subject's conscious perceptual experience of their causes*. Thus, as stressed in HHF, conscious perceptual experience is a form of explanatory hypothesis, that at once unifies, predicts, and inhibits the impingements that cause it, thereby minimizing the FE associated with them.

This task employs a vast range of the subconscious processing that underpins conscious experience. Thus, in reading a paragraph such as this, the reader's brain continually collects visual data, moving the reader's eyes in saccades that focus the most sensitive part of the retina on a series of areas about the size of a thumbnail on an outstretched hand. The focus thus skips from group to group of seven or eight letters, falling on each for about a fifth of a second, while the reader's brain/model continually unifies and predicts these partial scattered retinal snapshots as *conscious experience of understanding the thoughts of the author of the text*.

This continual collection of vanishing impingements at the sensory surface illustrates an “active” aspect of what Friston calls active inference. The inferences appear from the top level of the reader's conscious experience of understanding down through the neurocomputational hierarchy that underpins it. Thus, the reader *can see in the text what the author means*, without consciously attending to the way the author's paragraphs and sentences are compounded from words in accord with the syntax and semantics of the language in which she writes, or how these words are compounded of letters, or how the letters map to the combinations of sounds that enable the author's thoughts to map to the sounds of her language, nor finally to how these sounds relate to the grapheme fragments upon which the reader is focusing at eye level.

This illustrates some of the hierarchical predictive processing that FE neuroscience assigns to the circuity of the cerebral cortices. Roughly, such processing works continually top-down, bottom-up, and side-to-side, with each level in a cortical hierarchy passing predictions to the level below or beside, and each level below or beside passing prediction errors back to modify the hypotheses responsible for them, so as continually to minimize error over the whole cortical model of the causes of impingement at all levels of processing. This suggests that the levels or aspects of processing imagined in the last paragraph should be cortically realized in the same way. Since, again roughly, they also correspond to one or another theoretical domain in the cognitive/affective sciences of the mind, this indicates how the FE approach may prove particularly serviceable in integrating cognitive psychology and cognitive science more generally with neuroscience.

As Clark (2016, Ch 2) describes in detail, hierarchical processing in FE neuroscience is continually regulated by predictive assignments of precision. These “adjust the volume,” or synaptic gain, on hypotheses and data (PE's) over all levels of the hierarchy, amplifying or attenuating their causal role in processing. By this means the brain/model organizes coalitions of processing hierarchies for the explanation of data as the minimization of FE requires. Dopamine seems a potent neuromodulator in the optimization of precision (Iglesias et al., 2013), for example in attentional gain and the resolution of competing alternatives in action selection (Friston et al., 2014b; Schwartenbeck et al., 2015)—but with the consequence that aberrant assignments of precision (implementing aberrant salience) can produce both positive and negative symptoms of mental disorder (Kapur, 2003; Deserno et al., 2016; Howes and Nour, 2016). Comparably acetylcholine, maximally active in REM, seems important in regulating emotional memory via the amygdala (Jiang et al., 2016), and so for the role of dreaming in regulating emotion.

## Prediction, emotion, and dreaming consciousness

As discussed in Solms and Turnbull (2002), dreaming requires activation of the same dopaminergic SEEKING system as energizes purposive waking action, and that Panksepp and many others also take to be involved in delusion and other symptoms. Neuromodulation in sleep produces an attending away from sensory input by lowering the precision weighting of sensory prediction errors—to render perceptual accuracy irrelevant. This frees the brain to minimize complexity in REM dreaming, and so, implicitly, to resolve otherwise disruptive (emotional) conflicts. These processes too depend upon an appropriate modulation of precision or synaptic gain—and all seem likely to involve neuromodulatory transmitter systems, including the aminergic and cholinergic systems stressed by Hobson.

This implies, as HHF contend, that the kind of hierarchical precision-engineered processing that produces the conscious perceptual experiences of waking also produces the fictive conscious experiences of dreaming. But given the radical sensorimotor attenuation that attends REM, the FE-arousing data hierarchically processed in REM dreaming cannot derive from sensory impingement. A non-sensory source of FE-arousing data is required, and this would seem to lie in the powerful arousals of *emotion* that attend REM, which in turn seem part of the consolidation/reconsolidation of *memory*.

This accords with the tension in FE-minimization between accuracy and complexity sketched above. For insofar as emotions drive overly complex (overfitting) FE-minimizing responses to sensory PE in waking, then memories embodying such over-complex responses would themselves be a suitable target for FE-minimizing complexity-reduction in sleep and dreaming (and also in waking periods of attending-away from realistic response to perceptual impingement, such as in daydream, children's play, and other activities that Freud described in terms of phantasy). It also accords in interesting detail with Freud's (1974b, Ch VI) account of the *dreamwork* as operating to *condense emotionally significant elements* from a *range of memories associated with the dream*.

As we will illustrate below, the elements chosen for condensation apparently include both recent memories that are being consolidated and remote emotion-laden memories rearoused to be reconsolidated with them. What Freud describes as *condensation* would thus appear to reflect a *collection of data over remembered and associated arousals of amygdala-related emotion* that could be compared with the saccadic collection of sensory data in waking sketched above. Given *associated remembered arousals* as data, the fictive perceptual experiences of dreaming could serve to unify, predict, and inhibit these arousals, thereby minimizing FE (as emotional conflict/complexity) in a way closely analogous to the realistic perceptual experiences of waking.

This in turn would accord with “active systems” descriptions of memory consolidation (Rasch and Born, 2013; for an early version see Cartwright, 2010, and for a psychoanalytic anticipation Palombo, 1978). Sleep consists in stages of NREM (including SWS) followed by REM, with the latter characteristically lengthening, and the bizarreness of dreams often increasing, over the course of the night. Active systems accounts hypothesize that slow oscillations in SWS effect the downscaling required for synaptic homeostasis (Tononi and Cirelli, 2014), while hippocampal sharp wave ripples activate large areas of the cortex (Logothetis et al., 2012), transferring recent memories from the hippocampus to the cortical loci appropriate for their long-term storage (Staresina et al., 2015). As SWS yields to REM the reactivated memories are rendered increasingly plastic (Calais et al., 2015; Dumoulin Bridi et al., 2015; Ravassard et al., 2015), and associated amygdala-related emotions are rearoused, so that both can be reconsolidated under the impact of dreaming in a new and emotionally revised integration of emotion and declarative content (van der Helm et al., 2011; Genzel et al., 2015).

## Accuracy and complexity in waking, sleeping, and dreaming

Following Friston (2010) HHF describe how accuracy is maximized and complexity minimized over the wake-sleep cycle, and in a way that accords with the tension between accuracy and complexity, and the differing roles of waking sensory impingement and sleeping memory and emotion, sketched above. During a waking period sensory PE's entrain active inference, and perceptual learning operates to increase the accuracy of the model as it was prior to that period. This is essential for the accurate ongoing computation of sensory targets for waking regulatory action. (Helmholz, 1878/1971, p. 332) stressed that “each movement we make”—from the involuntary movements of the eyeballs and pupils in saccades through the planned and purposive sequences of movements by which we realize full-scale intentional actions and projects—can be thought of as an “experiment” by which we test the models that direct them in our brains. Thus, in waking our models accumulate the adjustments required for accuracy, embodying the increased complexity in synaptic connections that constitute a physiological load on the neurons involved.

Then during the precision engineered sensorimotor attenuation of sleep, processes including SWS, synaptic pruning, REM, and dreaming reduce the excess complexity, together with the synaptic connections in which it is embodied. As would fit such a two-stage process, we would expect the waking stage to maximize accuracy in a way that overshoots (overfits) the longer-term equilibrium, so that, waking accuracy assured, the sleeping stage can winnow back emotional complexity, and as hard as is required to optimize the model for the waking period that follows.

## Complexity reduction and memory consolidation

Such hypothesized complexity reduction in sleep would map directly to the “active systems” accounts of consolidation/reconsolidation above. Combining these with HHF would yield an account in which amygdala-related emotional rearousal and reintegration in REM and dreaming, as envisaged in Genzel et al. (2015), would be the final stage in both memory consolidation and complexity reduction, linking both with the increases in arousal and plasticity characteristic of REM. We could thus see synaptic homeostasis and emotional reintegration as rendering the posterior model of one waking period a simpler and better predictor for the next. And as indicated above, this theoretical unification admits further explication by psychoanalytic hypotheses relating to complexity, dreaming, and mental disorder.

## Realistic active inference in waking

In waking much minimizing of FE via action takes a familiar realistic form, in which PE's generate alternative parameters of emotion and/or desire for action that are entertained together with the alternative sensorimotor trajectories associated with them in affordance competition and action selection, and then—provided they generate trajectories that find their sensory targets—eliminated from ongoing neurocomputational work. Thus, suppose cells in the hypothalamus like those specified in Oka et al. (2015) respond to a predicted violation of homeostatic equilibrium by generating thirst and a desire to drink, and the brain responds by computing various motor trajectories that would slake this thirst.

Such trajectories terminate at sensory targets that Freud called *experiences of satisfaction*. For thirst this is the experience of drinking, and attaining the experience seems rapidly to inhibit both thirst and the interoceptive signals that generate it (presumably via proprioceptive feedback). In this the signal produced by the target experience acts rapidly, and well before the changes in the bloodstream that will secure homeostasis and inhibition over a longer period. Such an adaptation seems required for the rapid sequential satisfaction of desires in action.

## Fictive or counterfactual active inference in dreaming

As noted above, both intentional action and dreaming require activation of the dopaminergic SEEKING system, in which aberrant precision-weighting (salience) may also produce delusions and other symptoms. The simple example above enables us to see how such activation works in the sensorimotor attenuation of dreaming, for this is apparent in very simple dreams sometimes produced in response to interoceptive signaling in sleep. These make clear that such signaling yields what we can call *fictive* or *counterfactual* active inference, as opposed to the *realistic* kind that obtains in waking.

Thus, Freud reported that when he had eaten anchovies or some other salty food, he was liable to dream *that he was drinking cool delicious water*. After having this dream, perhaps several times, he would awake, find himself thirsty, and get a drink. Many people have had such a dream, and many are also familiar with its counterpart involving micturition. In waking from such dreams we intuitively regard them as caused by, and representing the satisfaction of, the desires we act on when we wake.

Active inference thus shows a common pattern between the realistic in waking and the counterfactual in dreaming. We can illustrate this in a simple diagram integrating some of the commonsense and neuroscientific notions involved. Abbreviating “desire” “belief” and “experience” by “des,” “bel,” and “exp,” and using P as a variable for which particular sentences that describe these in their relation to the environment can be substituted (Box 1).

BOX 1 **A comparison of the pacification or inhibition of desire by a conscious experience of satisfaction in waking action and in dreaming.**

**Figure:**
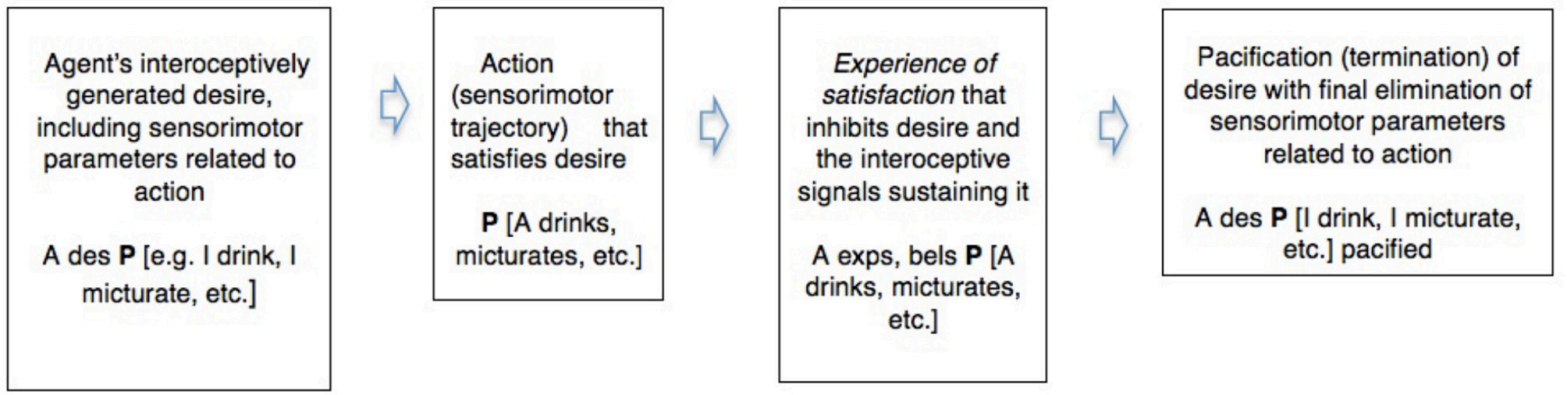
**Waking action (realistic active inference)**.

**Figure:**
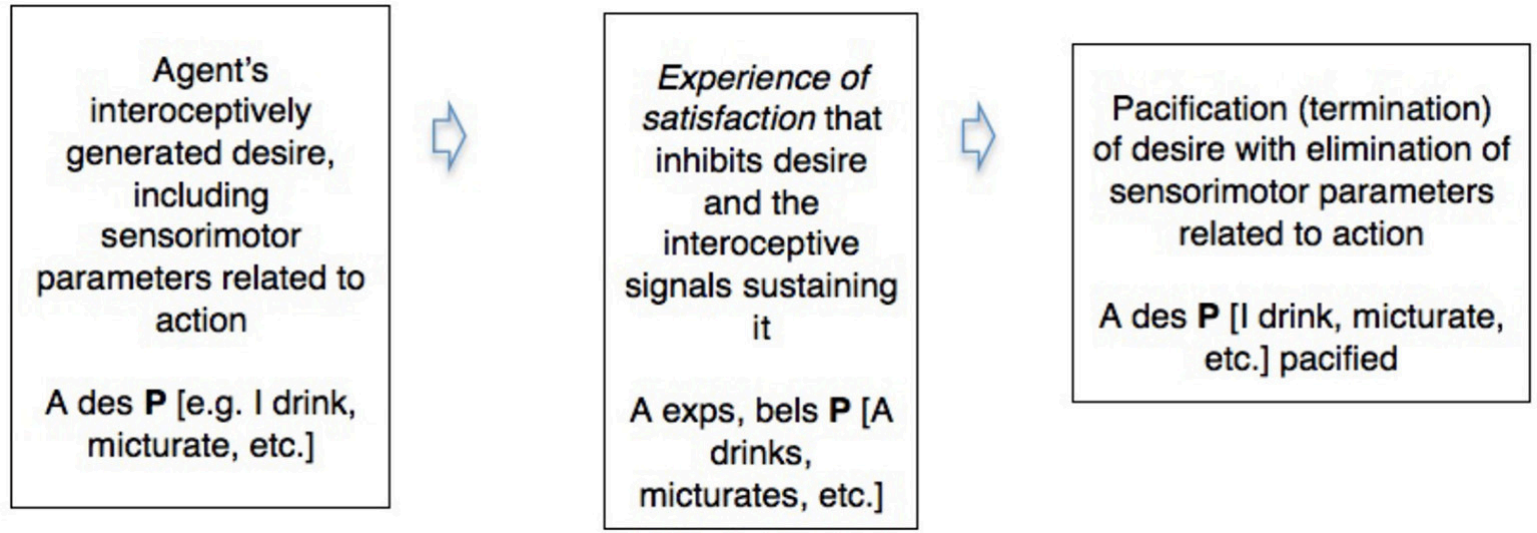
**Dreaming (counterfactual active inference)**.

This spells out, for a simple case, what Freud meant by claiming that dreams and symptoms are *wishfulfilments*, and also indicates how such fictions can inhibit interoceptive signals and their motivational derivatives like thirst or emotion, and thereby eliminate incipient kinematic parameters that might form part of a trajectory of waking action. And Freud also held that dreams exercise this counterfactual but genuinely parameter-eliminating role in the mitigation of *conflict*—in this case, the conflict between the desire for action and the continuation of sleep.

## Fictive or counterfactual active inference in waking mental disorder: a simple symptom in OCD

We can see how Freud compares dreams and symptoms by briefly considering the one case from which Freud's session-by-session notes partly survive, that of his patient known as the Rat Man (Freud, 1974c,d 155ff: hereafter R). His main symptom was a compulsion involuntarily to imagine, as in a waking nightmare, that his beloved father and venerated lady were being subjected to a terrible torture, in which *hungry rats ate their way into the anus of the victim, causing an agonizing death*. This first arose—precipitating a breakdown into what Freud called obsessional neurosis—when R felt forced to listen to the repellant story of the rat-torture, as told by “the cruel Captain,” his commanding officer on maneuvers, whom R intensely disliked for his fondness for physical punishment.

On hearing the story R had felt the earth move, as though a rat were tunneling beneath it, and imagined the torture being applied to his father and lady. Despite R's aversion to physical punishment, one might reasonably think that his repeatedly imagining people being tortured in the way the cruel Captain had described to him was a punitive expression of hostility toward the recipients of the torture. R did not imagine himself as administering the torture, but said it was done “impersonally.” But Freud found that his imaginings could be understood as stemming from unconscious impulsive rage, particularly toward his father, of whom the Cruel Captain had unconsciously reminded him in the episode that precipitated his breakdown.

R had already shown conflict related to rage in other ways. In his first consultation with Freud he described how when his venerated lady had left him alone to visit her grandmother he had felt “commanded” to cut his own throat with a razor, or again “to kill the old lady.” Although, R's diagnosis was clearly one of OCD, these commands seem instances of RAGE caused by PSG, in which the rage is also turned against the self, as by the Freudian superego. In the case of Elyn Saks, as we will see below, such anger gave rise to severe guilt and depression; and likewise R was anxious, guilty and depressed about his rat phantasies, saying at one point that he deserved to die for entertaining them.

As emerged in R's analysis, his “wishing the rats” on people was a regular expression of anger. In the preliminary meeting in which Freud told R his fee, R had thought (as he told Freud later) “For each *krone* a rat for [Freud's] children” (Freud, 1974d, p. 288); and this was the first of many such examples. Wishing the rats seemed also to be connected with physical punishment. In analysis R maintained that his father never punished him physically, but became increasingly fearful that Freud was liable to murderous rage, in which would beat him and throw him out the consulting room, or again fall on him like a beast of prey [or again like the rats of his phantasy] “to search out what was evil in him” (p. 285).

These manifestations of transference vanished when R remembered and re-experienced an episode from early childhood in which his father had angrily beaten him for urinating while lying between his parents in their bed. This fouling had apparently enraged his father, and R had felt in terror for his life. Although, R had forgotten this episode, his spectacular rage at his father while enduring the beating was a matter of family legend, and his father never punished him physically again. This immediately preceded the childhood onset of his early anxieties and depression about the likelihood of his father's death; and as R went over these things in his analysis, his “wishing the rats” evolved into a conscious expression of irritation that he used less and less, as he ceased to show the symptoms of OCD that had been related to it.

We can thus take R's symptom as instantiating a pattern similar to those above (Box 2).

BOX 2 **The pacification or inhibition of a desire involved in unconscious conflict by a conscious experience of satisfaction constituting a symptom.**

**Figure:**
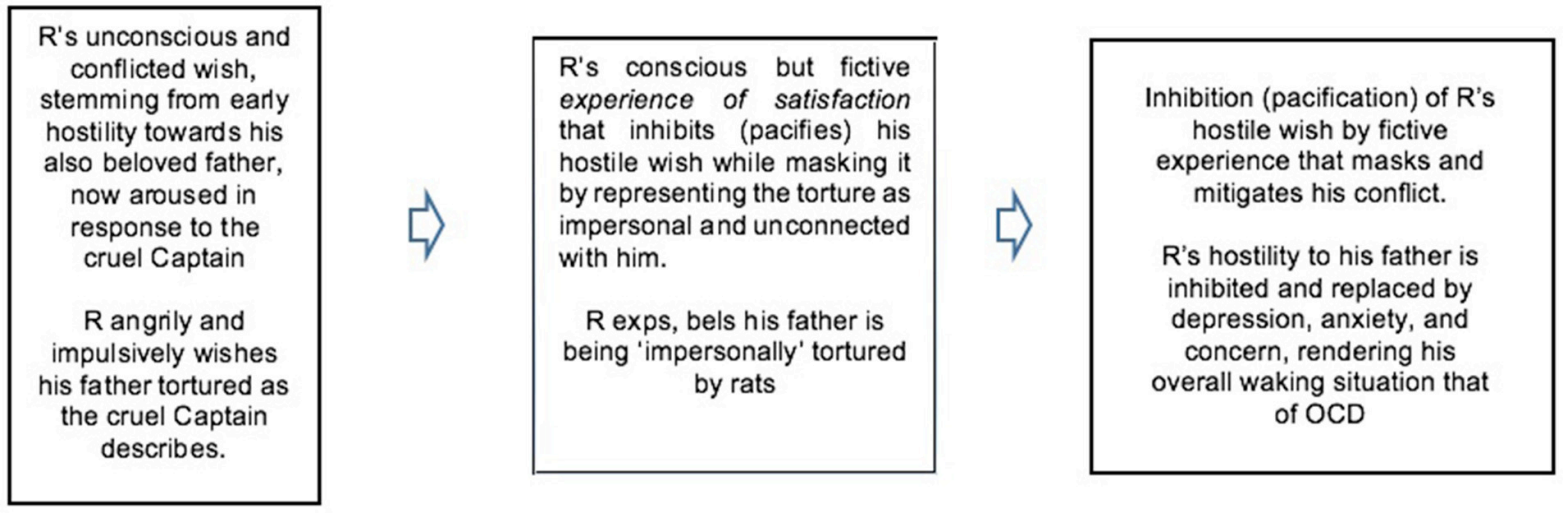
**R's symptom**.

As this makes clear, we can see R's symptom as produced by a dream-like condensation of his current anger at the punitive cruel Captain with his forgotten rage and fear toward his father—who was, therefore, the salient focus of the arousal of FE that his symptom (temporarily) served to reduce. (For relation of this to the psychoanalytic notion of repression see Hopkins, 2012). As this suggests, in both dreaming and disorder the input of FE to the hierarchical processes of complexity reduction by fictive experience would derive from memory and emotion, which must in any case also inform ongoing perceptual processing.

Here we also see a link between conflict, counterfactual inference, and complexity. When a fictive experience of satisfaction serves to mitigate a conflict, it does so by reducing or eliminating some of the conflicting parameters involved, as Freud's dream of drinking temporarily eliminates his nocturnal thirst, and R's imaginary torture temporarily eliminates the unconscious rage that conflicts with his love for his father, replacing it with anxiety and depression. The basic insight here is that conflict, which invariably has emotional or interoceptive aspects, can be equated with complexity in the statistical sense of FE neuroscience.

Such complexity is reported in waking by interoceptive prediction errors and quantifies the divergence between posterior (informed) and prior beliefs, where the latter would include the primary beliefs of infancy or, as above, emotion-laden priors dating from early childhood. From a technical perspective, this means that the resolution of conflict conforms to the statistical imperative to minimize complexity—and thereby normally renders internal models of the causes of sensory impingement better able to provide accurate and parsimonious predictive and regulatory explanations for the impingements themselves.

## Complexity and attachment

Since the regulatory functions of the generative model are built up in attachment and discharged via movement, we should expect the effects of excess complexity to show in relation to this process. As discussed in detail in Hopkins (2015), we can apparently see these regulatory functions in action in the “strange situation” procedure used to assess attachment from the end of the first year. This is conducted in a novel room (the strange situation: for further information see Howe, 2011) in which age-appropriate toys are provided to encourage exploration and play, and hence to activate the systems that Panksepp describes as SEEKING and PLAY. It turns on observation of the infants' reactions to short episodes (reduced in serious distress) in which (despite their angry protests) their mothers leave them alone or with a stranger. This apparently rouses PSG as well as FEAR of the stranger and RAGE toward the mother for inflicting such distress despite protests to the contrary.

Infants are classified as secure in attachment, or again as insecure—avoidant, ambivalent-resistant, or disorganized—depending on how they cope with these conflicts. Roughly, secure infants resolve the conflicts co-operatively, expressing their anger, distress, and fear as they occur, and on reunion accepting comfort and care, and so returning to exploration and play. Avoidant infants suppress the emotions, expressing them perhaps only in angry refusal to be comforted, and remain conflicted and stressed for a longer time. Those classed as ambivalent-resistant amplify the emotions, demanding closeness and comfort while also refusing to be placated; and they too remain longer in conflict and stress.

By contrast disorganized infants—who make up a large percentage of maltreated or abused samples, and are particularly liable to suffer later trauma-related disorders—are unable to adopt any of these overall strategies for coping with conflict. Rather they show various forms of *overt kinematic incoherence* in their behavior toward their mothers. These include contradictory behaviors, such as approach followed by avoidance, or combinations of the two; anomalous postures and mistimed movements; freezing or stilling; and signs of confusion.

That is: in this carefully standardized situation of emotional conflict, which reactivates conflicting parameters set earlier in infancy, the generative models of disorganized infants seize up, becoming incapable of predicting whether an optimal sensorimotor trajectory will approach or avoid their mothers. The only curves they are able to fit to their current data of perception and emotion here fail to yield a coherent social and spatial path. The complexity of affordance competition and action selection in this basic social decision is so magnified by conflict as to render the required computations too emotionally complex for their generative models to manage.

Here are two examples of such (hypothesized) excess complexity, from the behavior of a little boy and girl of 18 months, as described in Solomon and George (1999, p. 131).

In the second reunion Kate approached her mother with her arms outstretched…when she was about two feet away from making contact, she moved her arms to the side and abruptly circled away from her mother like a banking airplane. As she moved away she had a blank, dazed expression on her face.

In the first reunion, Sam approached his mother with his eyes cast down. When he was about two feet away he looked up at her, rising suddenly and making gasping noises with his breath as he did so. He quickly looked down again, bared his teeth in a half-grimace/half-smile and turned away. Hunching his shoulders and holding his arms and legs stiffly, he tiptoed to the other side of the room. He sat motionless in the chair for 30 s, grasping the armrests and staring straight ahead with a dazed expression.

This disorganization, moreover, bears comparison with that experienced in schizophrenia. Thus, consider Saks, (2008) account of her first experience of the latter, brought on by a critical remark from her father.

My heart sinks at the tone of his voice: I've disappointed him. And then something odd happens: My awareness (of myself, of him, of the room, of the physical reality around and beyond us) instantly grows fuzzy…I think I am dissolving …like a sand castle with all the sand sliding away in the receding surf. *This is scary, please let it be over!*…Most people know what it's like to be seriously afraid…“disorganization” is a different matter altogether…One's center gives way…

Of course, my dad didn't notice what had happened, since it was all happening inside me. And frightened as I was at the moment, I intuitively knew that this was something I needed to hide from him, and from anyone else as well (Saks, 2008, pp. 12–13).

Freudian hypotheses would explain these disorganizations, like that which struck R on hearing the cruel Captain, via emotional conflicts built into the self by the self-punishing superego/ego-ideal. But one need not adopt this theory to appreciate them as instances of excess complexity.

## Complexity conflict and trauma

As the foregoing indicates, complexity is conceptually linked with *emotional conflict* and *trauma*. Conflict involves the simultaneous activation of parameters producing inconsistent sensorimotor trajectories, such as we saw above; and these would be a clear target for complexity reduction in sleep. Again, complexity indexes the parametric changes required for *emotional learning* or *emotional adjustment to sensory impingement* (cf *affective load* in Levin and Nielsen, 2009); and experiences are rightly regarded as *traumatic* when, as in PTSD (Enlow et al., 2014) or BPD (Mosquera et al., 2014) the emotional adjustments (complexity) required for integrating them into thought and action are greater than the brain can manage.

Although, these forms of disorder, like disorganized attachment, are linked with traumatic abuse (Cyr et al., 2010), the work of Beebe and Lachman (2014) indicates something subtler. Their second-by-second microanalyses of face-to-face interactions between mothers and infants show that disorganization at 12+ months can be predicted from the 4th month by episodes of *emotional misrecognition* very different from abuse. These involve short (2.5 s) interactions in play, whose fine details are undetectable by unaided sight, and in which, among other things, the mothers concerned *refuse to recognize or co-ordinate empathically with their infants' expressions of distress*. Instead they respond with exaggerated surprise or smiling, or again, as distress increases, by facial freezing and/or looking away. Meanwhile the infants themselves—as in the later patterns of disorganization—respond with *rapidly conflicting expressions of emotion* e.g., whimpering and smiling within the same second or so; and they grow more distraught as no recognitional response is forthcoming. These infants seem frustrated not only by the original causes of their distress, but also in their desire that it be acknowledged and understood; and they respond with a short expression of the complexity/conflict that will later be expressed in approach/avoidance conflicts like those above.

We noted earlier that infants' expressions of distress seem to stem from the activation of the PAG, and in turn activate the PAG and other sources of conflict in mothers (Parsons et al., 2014; Dudek et al., 2016). These mothers are apparently so threatened by PAG-activating communications (or projections) of conflict and distress from their infants that they must block them entirely. This deprives the infants (as their mothers may have been deprived) of opportunities for learning in emotional interaction, and so for internalizing in their own regulative models the understanding responses of others. Instead they apparently learn that their conflicts and distress are unsharable, intolerable, and not to be recognized or known.

## Realistic and counterfactual active inference

The differences marked by the terms “realistic” and “counterfactual” thus derive from the distinct functions that active inference performs in the contrasting circumstances of waking and sleeping. All active inference involves selection among multiple competing (and hence so far counterfactual) possibilities for movement. But the function of waking regulatory control requires accuracy in the predictive hypotheses that implement it; hence hypothesis-selection in this context is constrained by PE toward realism.

By contrast complexity-reduction, whether in waking or sleeping, is discharged in respect of previously framed (remembered) hypotheses (including experiences of adverse conflicting emotions) whose complexity renders them unlikely candidates for realistic predictors in future. These would particularly include hypotheses (emotions and feelings) whose original parameters were *not* reduced or eliminated via their regulatory success, and so remained active prior to sleep. So inference relating to such past complex hypotheses would appear to compete with that required for ongoing perception-based regulatory control, as indicated by the relegation of perceptually unconstrained virtual-reality complexity-reduction to REM dreaming.

The observations about conflict and trauma above suggest that excess complexity (affective load) derives from these sources, and that the burden of complexity in infancy reflects the emotional adjustments required for establishing the bonds of attachment given the inescapable fact that parents or carers are the main salient causes of both gratification and frustration. This is exactly the kind of complexity that psychoanalysis finds expressed in the arousal of memory and emotion in both dreams and mental disorder; and accordingly we can see counterfactual active inference as healthy and functional in REM dreaming, but as constituting pathology when rearousals of conflict or trauma render it dominant in waking.

As the foregoing indicates, the complexity theory of disorder as advanced here is nearly a consequence of the account of complexity that informs HHF. For if the accumulation of complexity in waking is so serious a problem for the brain as to require complexity-reduction in sleep, then it would follow that inadequacies or malfunctions in such reduction – like prolonged sleep deprivation itself – might foster accretions of complexity that appeared in waking as mental disorder. In addition, just as the mechanisms of inflammation that have evolved to protect the body from injury can themselves cause bodily disorders, so the mechanisms that have evolved to reduce emotional complexity in sleep might cause disorders of the mind or brain.

## Complexity and psychosis

In order to illustrate this, let us briefly sketch some applications in schizophrenia, depression, and bipolar disorder. A first idea would be that schizophrenic and manic hallucinations and delusions are produced to perform the complexity-reducing function of dreams, but in waking (cf Llewellyn, 2011; Gerrans, 2014; Skrzypinska and Szmigielska, 2015). As an example we can consider the delusions developed by Elyn Saks, whose experience of schizophrenic disorganization was described above.

Saks entered psychiatric hospital in a savagely self-critical state which she kept repeating “I am a piece of shit and I deserve to die” (Saks, 2008, p. 61). Such self-hating internal conflict is a mark of the rage against the self that characterizes “introjective” depression (Blatt, 2004); and when antidepressants gave Saks some relief, she told her doctor that she felt less angry, and remarked on “how much rage I had felt, directed mostly at myself.”

Later, however, her self-reproaches returned in force, and her increasingly unbearable depression altered only when came to imagine herself “receiving commands” from “shapeless powerful beings that controlled me with thoughts (not voices) that had been placed in my head.” These commanded, e.g., “Walk through the tunnels and repent. Now lie down and don't move. You are evil.” She was also commanded to injure herself, which she did by burning herself with cigarette lighters, electric heaters, or boiling water, so that finally she spent most of her time “alone in the music room or in the bathroom, burning my body, or moaning and rocking, holding myself as protection from unseen forces that might harm me.”

These delusions fit the generalizations advanced so far. They served to mitigate the conflict, or reduce the complexity, of Saks' self-punishing depression, by replacing it with an imaginary relationship with punitive others. In framing them her generative model behaved as governments or nations (or other groups) often do, that is, shifting attention (precision) from intractable *internal* conflicts (and thereby reducing them) by focusing instead on imaginary *external* conflicts. This change from the internalization to the externalization of punishment constituted Saks' shift from depression to paranoia, and effected a temporary inhibition (reduction of parameters) of her internal self-punishment, and so a reduction in conflict (complexity) and FE overall.

Thus, and greatly simplifying, we can represent Saks's paranoia-like symptoms in a way parallel to the simple dream and symptom diagrammed above (Box 3).

BOX 3 **The pacification or inhibition of an intrapsychic conflict (between ego and superego) by a conscious experience of satisfaction constituting a projective delusion.**

**Figure:**
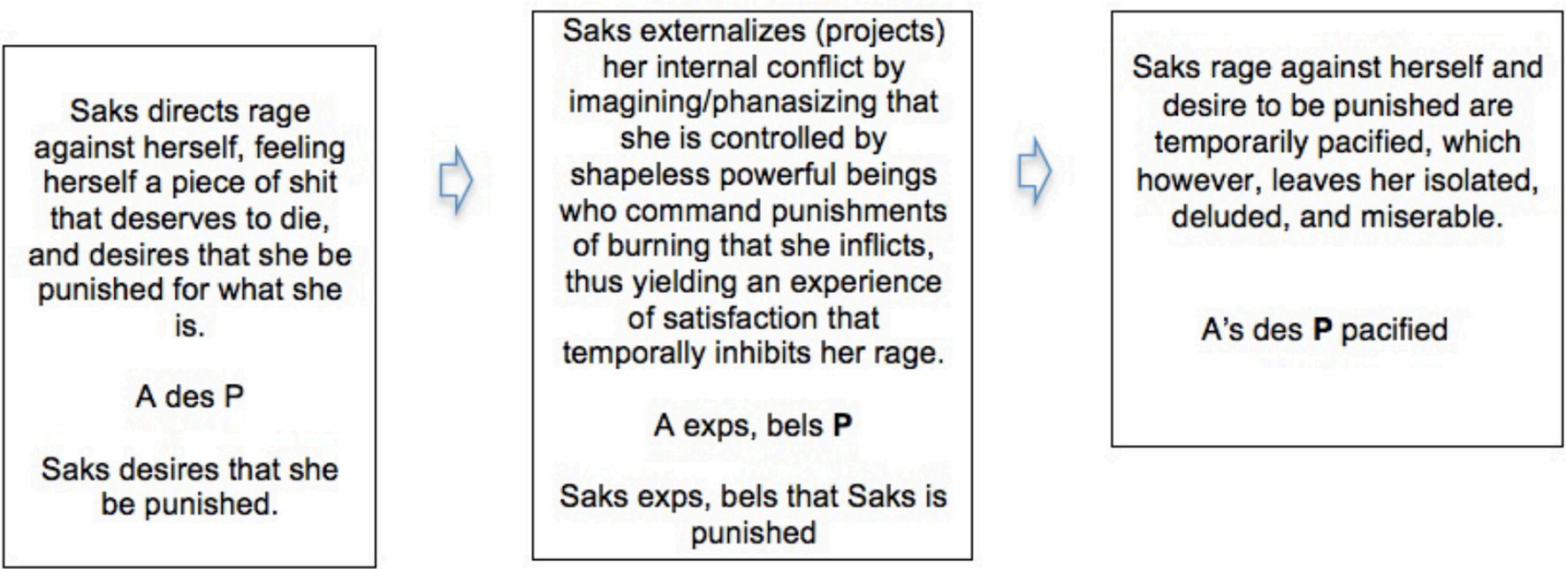

This kind of account dates from 1914, when Freud wrote that in schizophrenia, “the voices, as well as the undefined multitude [of imaginary critical psychological presences embodied in the superego/ego-ideal] are brought into the foreground again by the disease” so that the sufferer's superego/ego-ideal “confronts him in a regressive form as a hostile influence from without” (Freud, 1974e, p. 56). In such a case, while the reduction in internal conflict may relieve the unbearable internal hostility and depressive pain that can cause suicide, it also marks a deeper alienation from reality, and a deeper regulatory failure on the part of the generative model.

In constantly berating her as a piece of shit who deserved to die Saks' self-critical faculty was already punishing her dysfunctionally, for some imagined or phantasied transgression; so her resorting to counterfactual active inference to externalize the punishing agency in the “shapeless powerful beings” of her delusions was a *further* step from reality. This is why the externalization constitutes a deeper regulatory failure; why Freud describes it as regressive in the quotation above; and why such paranoia involves a deeper alienation from reality than depression—even though a main risk in schizophrenia is suicide in the depressive phases in which the subject is attempting to re-establish internal regulatory control.

Freud remarked that R's relief from his OCD came with his recognizing *himself* in the invading rats of his phantasy; and we can now see that this symptom, like Saks' delusions, effected an imaginary relocation (projection) of an angry (and biting) part of himself into another. The oscillation between depression and mania in bipolar disorder shows a comparable mechanism, in which attempts to maintain internal but overly depressive regulatory control give way to delusions in which, e.g., the subject expects and imagines himself accomplishing wonderful things, and the SEEKING system is again overactive in a way admitting control by dopamine antagonists such as haloperidol. A third complexity-reducing response, discussed in detail in Watt and Panksepp (2009) and Alcaro and Panksepp (2011), is the inhibition of the SEEKING system itself, as seen in the response to PSG in infant animals. (This reduces FE from sensory impingement in a way comparable to the “dark room” often posed as a problem for Friston's account.)

## Waking disorder and complexity reduction in sleep

As these sketches illustrate, the complexity theory integrates the full range of psychoanalytic hypotheses about dreaming, development, and disorder into the perspective of FE neuroscience, where they might receive additional testing. But the theory also implies something further, namely that we should understand mental disorder in terms of *systematic* connections between failures/malfunctions of complexity reduction in sleep and the emergence of symptoms in waking life.

These are clearly visible in PTSD, and the nightmares that abort dreaming are now themselves regarded as causes of waking symptoms, including suicide (Littlewood et al., 2016). More generally, a recent study found that 55% of individuals diagnosed as psychotic (as opposed something less than 5% of the general population) had nightmares at least once a week, and that the emotional severity of these disturbances of sleep was correlated with that of their waking symptoms (Sheaves et al., 2015; see also Levin and Nielsen, 2009).

Nightmare seems best understood as produced by the arousal of fear or panic in the consolidation/reconsolidation of memory that coincides with complexity reduction in sleep. As illustrated below, complexity reduction in dreaming often begins with a phase expressing the conflicts that the dream will go on to mitigate; and in nightmares (such as that by Obama discussed in Hopkins, 2013) this initial expression causes premature waking. Thus, both psychoanalysis and systems consolidations accounts can agree in regarding nightmares as failed attempts at the kind of emotionally pacifying amygdala-related processing hypothesized in active systems consolidation above. The correlation between the emotional severity of the nightmares and the waking symptoms suggests, consistently with the complexity theory, that *both* the sleeping and waking disturbances have a common source in aversive emotional arousal related to memory, and also that the persistence of the waking symptoms is related to failures in complexity reduction in sleep.

A range of waking disorders, including schizophrenia, mania and depression, vary systematically with less obvious disorders in sleep, involving abnormalities in SWS, REM, and the way these relate to dreaming. As the complexity theory would suggest, disorder in sleep seem often to predict that in waking (Krystal, 2012; Reeve et al., 2015; Zanini et al., 2015); and cognitive bizarreness tends to correlate across disorders as between dreaming and waking. Thus, normally bizarreness in dreaming greatly exceeds that in waking. In depression, by contrast, dreaming, when it occurs, is often mundane, emotionally flat, and lacking in bizarreness (Beauchemin and Hays, 1996), so that cognitive bizarreness in dreaming and waking tend toward a level. Something similar appears in schizophrenia and mania, in which both dreaming and waking bizarreness are elevated (Cavallotti et al., 2014).

Cartwright (2010, Ch. 4; see also Nutt et al., 2008; Palagini et al., 2013) describes how severe depression often involves a premature transition from early light sleep to a disordered form of REM. This omits deep SWS in which growth hormone is released, and memory transfer from the hippocampus to the cortex normally takes place. The premature REM is abnormally sparse or dense, coming in “eye movement storms,” accompanied not only by high limbic/paralimbic arousal but also by high (and likely conflict-controlling) activity in frontal executive areas. In consequence many severely depressed patients apparently *do not* dream in REM.

Taken together with the mundaneness of dreaming in less severe depression, this suggests that the bizarreness of dreams partly reflects the complexity aroused and available for reduction in them. This would accord with Kirov's (2013) suggestion that complexity reduction takes place periodically during successive NREM-REM cycles over a night, with each cycle potentially broadening and/or deepening the work of consolidation/reconsolidation of its predecessors. Since the sources of complexity in remote memory are intrinsic and inexhaustible, increases in bizarreness during a night would mark ongoing successful reduction; and the bizarreness itself would partly reflect an increasing use of *condensation*, as discussed below.

By contrast disturbances that abort complexity-reduction in dreaming seem also, as in PTSD, to give rise to symptoms in waking. As shown by Vogel et al. (1968, 1977, 1980) symptoms of even severely depressed individuals are altered without medication by 3 weeks of carefully controlled deprivation of REM sleep. This causes an REM rebound in which SWS and REM are restored to a more normal balance and symptoms are relieved. This illustrates the leading role that the complexity theory assigns to processes that have evolved to reduce complexity in sleep; and so do Cartwright's further observations as to the way the quality of patients' sleep and dreaming predicts whether they will recover from depression. (For related recent research incorporating a psychoanalytic perspective see Fischmann et al., 2013)

A comparable situation holds in schizophrenia, the disorder held most closely to resemble dreaming (Llewellyn, 2009, 2011), and in which dreams are often impoverished or short but related to waking symptoms (Chang, 1964; Carrington, 1972; Lusignan et al., 2009; Noreika et al., 2010; Mota et al., 2014). In healthy individuals the slow waves, spindles, and sharp-wave ripples that implement the transfer of memories to the cortex during the consolidation/reconsolidation of memory are tightly synchronized. In individuals diagnosed as schizophrenic they are not (Gardner et al., 2014). Slow wave activity in sleep—which as Cartwright (2010) notes may also be associated with sleepwalking and night terrors—is abnormal and reduced. This goes with an *increase* in slow wave activity in waking, particularly when the disorder is active, and also with abnormal dopamine release (Slifstein et al., 2015), blunted in prefrontal but increased in striatal areas. As Duan et al. (2015) and Lisman (2016) contend, this may impede prefrontal working memory; and since striatal dopamine can also cause delusions, this may also be a source of delusions such as Saks' above.

Friston and his colleagues distinguish *dysconnection*, the dysfuncional integration of distributed neuronal processes, usually at the synaptic level, from *disconnection*, the anatomical disruption of long-range connections at the level of white-matter tracts and axonal processes (Friston et al., 2014a). Recent work on dysconnection (van den Heuvel et al., 2015) suggests that both may be due to the loss of dendritic spines on cortical neurons, which may in turn be caused by the removal of calcium-permeable AMPA receptors in sleep (Lanté et al., 2011; on mechanisms of pruning see also Gonçisalves et al., 2016). Such losses have long been documented in schizophrenia (Glausier and Lewis, 2012) and more recently in bipolar disorder (Konopaske et al., 2014). Since the 1980's neuroscientists have speculated that this is due to synaptic pruning (Feinberg, 1982; Catts et al., 2013), and this has now received striking genetic support (Sekar et al., 2016).

Accordingly Cannon (2015) argues that synaptic pruning is a key explanatory factor in the development of schizophrenia; and the same might hold, although in a different form, for depression (MDD), biolar disorder (BP) and others. This suggests, in accord with the complexity theory, that nocturnal pruning can take aberrant forms that render individuals like Elyn Saks less able to process conflict or trauma in sleep and dreaming, and so in waking to require the excessive recourse to phantasy or delusion that apparently constitutes the disorder. Moreover, as Cannon (2015) reports, it seems that circuit alterations wrought by synaptic pruning might themselves produce the altered dopamine release that, as noted above, may facilitate both delusion and “negative” symptoms in schizophrenia (and would have effects in other disorders as well); and mouse models (Kim et al., 2015) may support this.

Synaptic and axonal degradation can of course have other causes related to complexity, such as the adrenal steroids produced by stress associated with conflict and trauma, as emphasized in Kandel (1999) and Altena et al. (2010) report that such degradation is also caused by insomnia. The patterns of deficit related to schizophrenia and depression, as described in Schultz et al. (2012), Grieve et al. (2013), and Gong et al. (2015), seem to overlap with areas known to be involved in regulating emotion and conflict (Etkin et al., 2015). This, again, might extend to a number of disorders that psychoanalysis explains in terms of trauma, conflict, and phantasy, but current psychiatric classifications describe as at once very diverse but also liable to unexplained co-morbidities and overlapping of symptoms. For as Goodkind et al. (2015) report, a range of Axis 1 mental disorders seem related to such patterns of reduction.

However produced, these patterns can be seen, consistently with the complexity theory, as indicating a reduced capacity on the part of the generative model to cope with trauma and conflict, and a greater reliance on varying forms of phantasy or virtual reality in doing so. Thus, both the commonalities and the varieties in a range of mental disorders and everyday problems in living may be explicable in terms of emotional conflict or complexity and the factors that have evolved to reduce it.

## Complexity reduction in REM dreaming

Dreams could evidently play the complexity-reducing role sketched above during the consolidation/reconsolidation of memory, and as already stressed this would fit with recent work on REM dreaming as performing “emotional valence re-evaluation and adjustment” in updating and revising the amygdala-related emotions associated with memory (van der Helm et al., 2011; Genzel et al., 2015 see also Gujar et al., 2011; Goldstein and Walker, 2014; Hutchinson and Rathmore, 2015). We would expect complexity reduction to occur together with the updating of the emotional significance of old memories, and Soeter and Kindt (2015) have recently provided powerful evidence of the plasticity of fear memory under reconsolidation. This suggests that the vivid experiences of dreaming in the apparently plasticity-inducing circumstances of REM could have a powerful revisionary effect on the emotional significance of the whole family of cortically embodied long-term memories undergoing updating in sleep, while at the same time strengthening (and without distortion) their declarative content.

This would provide for the maintenance of accuracy together with the reduction of emotional complexity; and it would also correspond almost exactly with the understanding of the role of dreaming in the consolidation of memory that is to be extracted from Freud. To appreciate this we can observe that Freud's analyses of dreams characteristically contain, among others, three kinds of elements. These and their relations are discussed in more detail in Hopkins (2015), and include the following

- *The consciously remembered dream*. The sequence of episodes that make up the dream, or what Freud calls the *manifest content* of the dream.
- *Conscious memories and feelings from the day of the dream*. These include the episodic memories that, according to active systems accounts, are under consolidation in the dream, and so are the emotionally significant new memories that are under transfer to the cortex from the hippocampus during the SWS that precedes the REM in which the dream in (i) occurs. Thus, it seems that in the dream itself these new memories are being consolidated and emotionally integrated with the rearoused cluster of memories and emotions (iii, below) already stored in the cortex.
- *Deeper memories and emotions* (as well as thoughts, feelings, wishes, etc.) *systematically related to (i) and (ii)*. These *remote memories* and *significant emotions* are part of what Freud calls the *latent content* of the dream, as collected in his process of free association. In that context they can be seen to relate to (i) and (ii) in such a way as to provide evidence (similar to that in the simple instances of dreaming above) for the hypothesis that *these are the deeper memories and emotions that were unconsciously aroused in active inference on the previous day*, and hence also *the memories and emotions that systems consolidation accounts take to be rearoused and reconsolidated during REM and dreaming*. They are thus also the main targets for the *complexity reduction* taking place, as HFF hypothesize, in REM dreaming, and will thus serve as more efficient predictors in the next waking period.

Psychoanalytic and system consolidation accounts of (REM) dreaming are thus closely related both to one another and to the HHF account of dreaming as complexity reduction. We can summarize this by saying that Freud's conception of *the latent content of a dream* encompasses *the same cortically stored memories and associated emotions* as the systems accounts take to be aroused for reconsolidation and emotional revision under the impact of Freud's *manifest content* in REM.

Thus, both Freudian and systems consolidation accounts can regard the manifest dream experience as caused by the arousal of the same mainly unconscious memories and emotions that were active on the day of the dream; and both regard the manifest dream as working to alter some of these emotions during their arousal. The alterations proposed by both accounts, moreover, are such as would reduce emotional complexity (including conflict and potentials for trauma) in the sense we have been considering.

Taken in combination these three accounts—psychoanalytic, systems consolidation, and complexity reduction—converge not only on the memories and emotions whose arousal causes dreaming in REM, but also on the nature of the emotional changes that the fictive experiences of dreaming produced by counterfactual active inference in REM function to bring about. This in turn indicates that we can further integrate neuroscience and depth psychology by understanding the nature and specifics of complexity reduction in the actual cases in which psychoanalysis enables us to trace the memories and emotions in question and describe instances of alteration and its effects.

To see this briefly but with some detail let us consider the combined accounts as related to a single element of the dream Freud first analyzed. In this dream Freud met his former patient Irma, who introduced the topic for complexity-reduction by complaining to him about her continued suffering after her therapy. He examined her together with Dr. M, the leading figure in their medical circle, and other members of the circle who materialized in the dream. M confirmed Freud's conclusions, and the examination made all present aware that Irma's continued suffering was caused by a thoughtless injection of the toxic chemical trimethylamin, administered shortly before by another member of the circle, Freud's friend and family doctor Otto.

Thus, here is (part of) Freud's description of examining Irma in the dream (Freud, 1974b, p. 107), with the particular element we will be considering emphasized in bold:

I took [Irma] to the window [to examine her] and looked down her throat…I at once called in Dr. M., and he repeated the examination and confirmed it…[saying] “There is no doubt it is an infection”…We were directly aware, too, of the origin of her infection. Not long before, when she was feeling unwell, my friend Otto had given her an injection of…trimethylamin…***One does not make injections of that sort so thoughtlessly***…And probably the syringe had not been clean.

This imaginary episode was clearly related to two sets of memories and emotions, as described in (ii) and (iii) above. The first derived from the day of the dream, when Otto had called on Freud and mentioned that he had just visited Irma and her family at their summer place, and while there had been called away to give someone an injection. Freud asked how Irma was, and Otto said that she was “better, but not yet well.” On hearing this Freud felt vaguely annoyed, as if Otto were impugning his treatment of Irma; and that evening he sat writing out Irma's case history until late at night, to show to M, a leading figure in their medical circle, to justify himself.

These memories are clearly concerned with Freud's feeling of being criticized by Otto and his wish to justify himself; and Freud's discovery in the dream that Otto's thoughtless injection was responsible for Irma's continued suffering provides both justification and grounds for retaliatory criticism. So we see a connection between the manifest content and the emotions of the day that is similar to those in the simple examples above. Just as Freud's dream of drinking and the desire to drink we take to have caused it are related as desire and experience of satisfaction, so Freud's experience in this more complex dream can be seen as fictively satisfying the desire to justify himself and the annoyance with Otto that he felt on the evening of the dream. So here, as before, we naturally take the dream experience as both *caused by*, and also as *serving to pacify* (that is, to *inhibit*) the emotions aroused in REM.

In this account the complexity of Freud's model as he went to sleep mainly reflected the parametric changes produced by the small but genuine trauma of this unpleasant and potentially depressing experience, and his writing up Irma's case history until late that night suggests emotional overfitting on this topic, due to his remote but still active memories connected with it. Hence the construction in this dream of a radically counterfactual *experience of satisfaction*—combining assurance of his medical competence with innocence and vindication against Otto as regards the thoughtless medical misuse of toxic substances—was calculated to produce a prior-restoring (and hence complexity-reducing) alteration in emotion that would render his model optimally prepared for action-directing work on the day to come.

These reflections are strengthened by considering the remote memories and emotions that are part of the latent content of the dream. We can see how Freud's free associations led to these by starting with those to the element on which we have focused.

*One does not make injections of that kind so thoughtlessly*…this sentence in the dream reminded me once more of my dead friend who had so hastily resorted to cocaine injections…I noticed too that in accusing Otto of thoughtlessness in handling chemical substances I was once more touching upon the story of the unfortunate Mathilde, which gave grounds for the same accusation against myself…(Freud, 1974b, p. 106).

These associations go directly to remote memories freighted with guilt and shame: to Freud's own involvement, as a physician pledged to *primum est nihil nocere*, in the advice and administration of injections that proved lethal. For as Freud says, they indicate that *he felt himself guilty of the kind of thoughtless handling/injection of toxic substances of which he accused Otto in his dream*.

Freud had been an early advocate of the medical use of cocaine, and his enthusiasm—which extended to arguing, on the basis of his own experience, that cocaine was not addictive—“had brought down serious reproaches on me.” During this period a beloved friend and mentor, the talented scientist Ernst Fleichel von Marxow, had become addicted to morphine, taken to relieve incurable pain from an infection and amputation resulting from dissecting a cadaver. Freud had recommended that von Marxow use cocaine as a supposedly non-addictive substitute for morphine. He rapidly became addicted and the decline that followed caused Freud great grief and guilt.

The “unfortunate Mathilde” was another about whom Freud had reason to feel intense guilt. He had given her injections of sulphanol, considered harmless up to that time, and she too had died as result. This death, moreover, was associated in Freud's mind with the fate of his own daughter, who had also been seriously ill.

My patient—who succumbed to the poison—had the same name as my eldest daughter. It had never occurred to me before, but it struck me now almost like an act of retribution on the part of destiny. It was as though the replacement of one person by another was to be continued in another sense: this Mathilde for that Mathilde, an eye for an eye and a tooth for a tooth (Freud, 1974b, p. 111–112)

In considering how his associations had led to these topics, Freud remarked that it seemed he “had been collecting all the occasions which I could bring up against myself as evidence of lack of medical conscientiousness.” With hindsight we can see that this was the activity of the part of himself that he would later call the superego, which not only accused him with his worst medical derelictions but also threatened him with retribution for them in the form of the death of his own daughter. This was the unconscious version, in Freud, of the kind of conflict with the superego consciously expressed in Saks' berating herself as a piece of shit who deserved to die. And as with Saks, it involved activity of a part or aspect of the self being experienced as coming outside—not from shapeless powerful beings such as Saks described, but as *retribution on the part of destiny*.

Thus, we can diagram the complexity-reducing function of Freud's dream as follows starting from the initial stage of memory consolidation in SWS (Box 4).

BOX 4 **An initial stage of memory consolidation: Freud's memory of an emotionally significant experience from the day is transferred to the cortex for long-term storage during SWS.**

**Figure:**
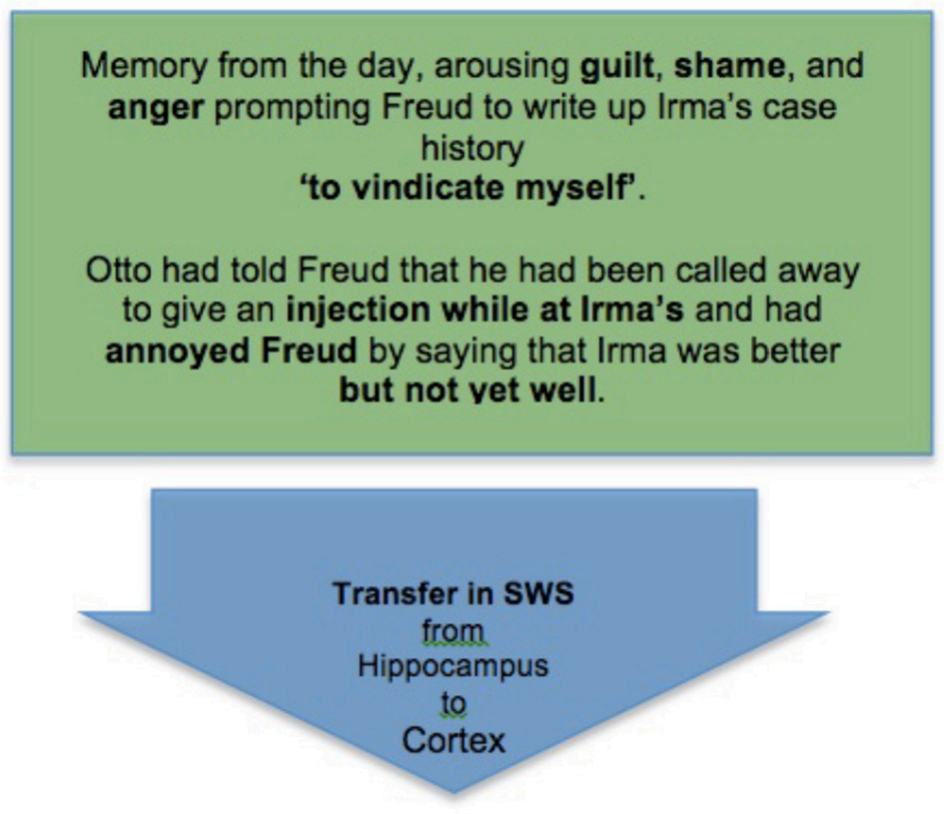

This is followed by the second stage, of arousal of memory and emotion in REM, as understood in light of the memories recovered by Freud in free association (Box 5).

BOX 5 **A second stage in memory consolidation: The cortically stored memories which gave Freud's experience of the day its particular emotional significance are re-aroused in REM so that the new memory can be consolidated together with them as part of a complex that has been emotionally revised (simplified) in light of the day's experience.**

**Figure:**
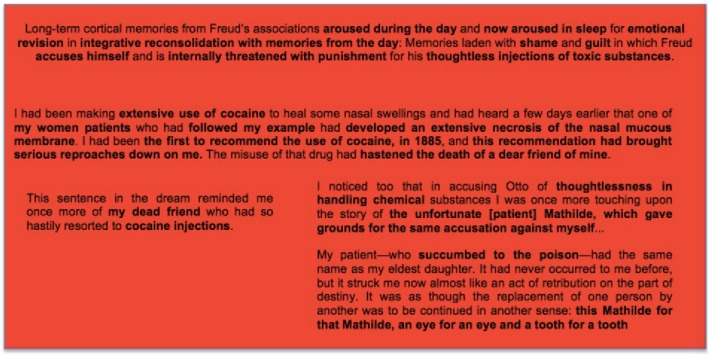

And finally we have the work of counterfactual active inference in response to these arousals, inhibiting (pacifying) them by creating Freud's fictive experience of innocence and vindication, and so reducing the emotional complexity (adversity, conflict, and potential to cause depressive trauma) of the whole family of memories under reconsolidation (Box 6).

BOX 6 **A final stage in memory consolidation: The complex of memories and emotions rearoused in REM produce a series of emotionally simplifying dream experiences, which enable the complex to be reconsolidated in a less complex (less averse, conflicted, and trauma-producing) form.**

**Figure:**
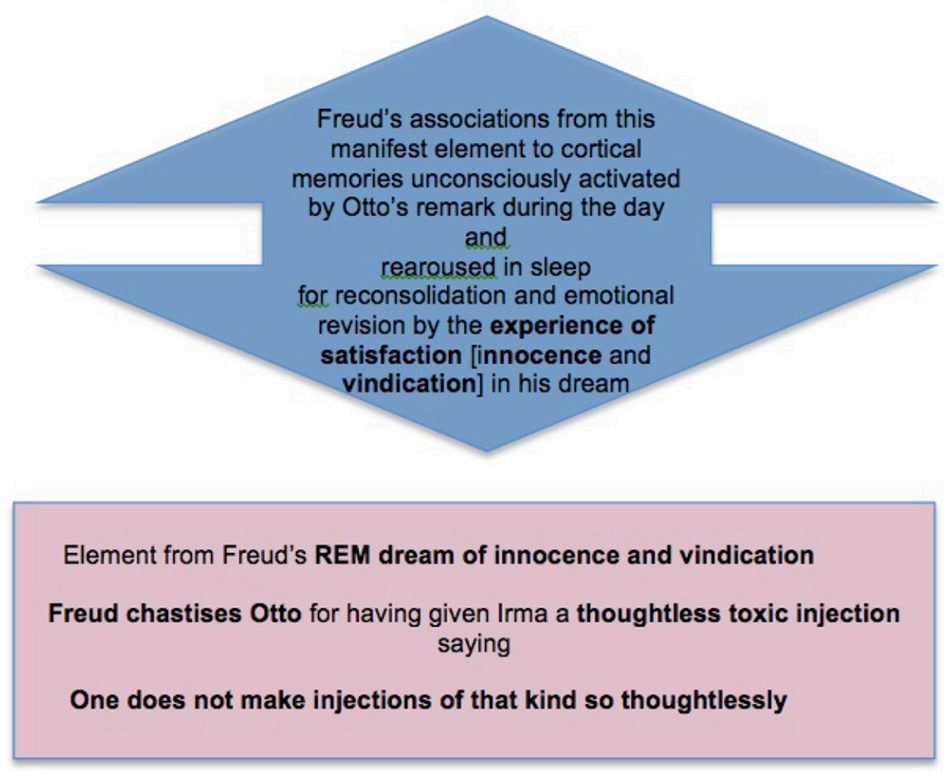

## Condensation and symbolic cognition

Above we noted that Freud described the *dreamwork* (Freud, 1974b, Ch. VI) as *condensing* emotionally significant elements from a range of associated memories, including recent memories under consolidation and remote memories rearoused for reconsolidation with them. In this example we can see such condensation clearly in the figure of Irma, whose role as recipient of Otto's toxic injection in the dream condenses her with the “dead friend” and “the unfortunate Mathilde” in Freud's associations. This *condensation* was a form of *symbolization*, bridging Freud's recent upsetting memory of Otto's remark about Irma with his temporally remote memories about the friend and patient who were the deeper sources of his guilt and shame, and preparing for the emotional whitewash provided by the rest of the dream.

This condensation enabled Freud's *fictive conscious experience of innocence in respect of a toxic injection given to Irma* to extend its inhibitory reach to his real remembered arousals of emotion about his role in toxic injections, and in a way that is structurally comparable to the role of consciousness-constituting FE-minimizing inference in waking. Symbolic cognition thus also plays an important role in the minimization of FE, and Hopkins (2000, 2015) describe how the dreamwork in this example makes copious use of symbolism that can be discussed as conceptual metaphor. Malinowski and Horton (2015) discuss conceptual metaphor as part of symbolic embodied cognition in an account of emotion-assimilation in dreaming that also relates it to condensation and memory.

It thus appears that a key role of active inference in the construction of dreaming consciousness is to inhibit and alter adverse and conflicting emotions, so as to reduce or eliminate the overly complex parameters/states introduced in their waking arousal. We can see Freud's dream as accomplishing this in a way akin to Bayesian model selection (cf Fitzgerald et al., 2014). The dream introduced an *ideal alternative counterfactual model* which perforce realized *an ideal fictive sensorimotor trajectory* by which, and in accord with Freud's priors of the previous day, the adverse emotional situation precipitated in him by Otto's unwelcome remark could be restored to a personally satisfactory (and free-energy minimal) equilibrium. Thus, Freud could awake in the morning free from the self-justificatory and depressive trend in which he had spent the day and evening after Otto's remark, and address his problems as a physician and psychiatrist via the creative activity of analyzing his own dream, thereby altering his own theories and practice for the better.

In addition we can see the fictive construction in Freud's dream to have played a role comparable to that in Saks' delusions. In both cases the self was threatened by conflict in the form of severe and depressing self-reproaches, and in both this conflict was mitigated by the creation of a counterfactual virtual reality that externalized the conflict. In Saks the moralistic agency of conflict (the superego) was externalized by projection into the “shadowy, powerful beings” who commanded her to undergo self-tormenting penance. In Freud, this agency remained intact and functioning, but the counterfactual virtual reality, as in a manic episode, directed its critical functioning away from Freud himself and on to Otto instead. Thus, Freud's dream relieved his internal conflict while keeping his internal locus of self-regulation intact and exercised, whereas Saks' delusions fragmented that locus and thereby deepened her illness.

The discharge of this complexity-reducing function evidently requires the collection/organization of emotionally aversive memories, apparently effected in the transfer from hippocampus to the cortex referenced above. As other disturbances in SWS also suggest, this transition seems liable to malfunction. As noted above in nightmares the arousal of aversive memories and emotions renders the dreamer/model incapable of devising any ideal alternative. Again, Saks' depression and paranoia both apparently result from an accumulation of self-directed aversive emotion, caused, we may hypothesize, by destructive phantasies (e.g., those of killing babies discussed in Hopkins, 2013) that made her guilty.

Dahan et al. (2007) report that a kind of “burst firing” of VTA dopamine neurons occurs in both REM and waking consummatory reward. Such firing has recently been modeled as modulated by acetylcholine, which is elevated in REM (Knudstrup et al., 2016). It would fit the account here if the complexity-reducing power of wishfulfilling counterfactual virtual reality in REM dreaming derived from such aminergic/cholinergic engineered precision. (An additional possibility is that this is linked with the reversal of hippocampal theta oscillations, Jackson et al. (2014) that Genzel et al. (2015) speculate may “provide the milieu” for the revision of emotional memory in REM and dreaming).

## Conclusion

We have seen how Freud's speculative FE neuroscience relates to the rigorous and potentially unifying paradigm now advanced by Friston and colleagues. This in turn enables us to see how the statistical conception of complexity employed by Friston relates to emotional conflict and trauma; how symptoms as well as dreams can be understood in terms of complexity-reduction; how REM dreaming can reduce complexity in the consolidation/reconsolidation of memory; and how complexity and the mechanisms that have evolved to reduce it seem pivotal for the understanding of mental disorder. This linking of complexity, dreaming, and disorder also indicates that Freud and free association offer a distinctive path—but one consilient with cognitive science, FE neuroscience, and computational psychiatry—toward understanding them together. Fuller interdisciplinary co-operation along this path might well make a significant contribution to further progress.

## Author contributions

The author confirms that he is the sole contributor to this work and approves it for publication.

## Funding

This work was not supported by a funding body.

### Conflict of interest statement

The author declares that the research was conducted in the absence of any commercial or financial relationships that could be construed as a potential conflict of interest. The reviewer KF declared a shared affiliation, though no other collaboration with the author to the handling editor, who ensured that the process nevertheless met the standards of a fair and objective review.

## Footnotes

^1^For current web resources on these topics see https://en.wikipedia.org/wiki/Free_energy_principle and http://ilab.usc.edu/surprise/. The term “complexity theory” was introduced by Chris Mathys at the 2013 Free Energy conference in Berlin, and used in our joint presentation at the 2014 Neuropsychoanalysis conference in New York.
